# Theoretical Design of Multilayer Dental Posts Using CAD-Based Approach and Sol-Gel Chemistry

**DOI:** 10.3390/ma11050738

**Published:** 2018-05-07

**Authors:** Saverio Maietta, Roberto De Santis, Michelina Catauro, Massimo Martorelli, Antonio Gloria

**Affiliations:** 1 Department of Industrial Engineering, Fraunhofer JL IDEAS—University of Naples Federico II, P.le Tecchio 80, 80125 Naples, Italy; smaietta@unina.it (S.M.); massimo.martorelli@unina.it (M.M.); 2 Institute of Polymers, Composites and Biomaterials—National Research Council of Italy, V.le J.F. Kennedy 54—Mostra d’Oltremare Pad. 20, 80125 Naples, Italy; rosantis@unina.it; 3 Department of Industrial and Information Engineering, University of Campania “Luigi Vanvitelli”, Via Roma 29, 81031 Aversa, Italy; michelina.catauro@unicampania.it

**Keywords:** computer-aided design (CAD), mechanical analysis, finite element analysis (FEA), composites, hybrid materials, biomedical applications

## Abstract

A computer-aided design (CAD)-based approach and sol-gel chemistry were used to design a multilayer dental post with a compositional gradient and a Young’s modulus varying from 12.4 to 2.3 GPa in the coronal-apical direction. Specifically, we propose a theoretical multilayer post design, consisting of titanium dioxide (TiO_2_) and TiO_2_/poly(ε-caprolactone) (PCL) hybrid materials containing PCL up to 24% by weight obtained using the sol-gel method. The current study aimed to analyze the effect of the designed multilayer dental post in endodontically treated anterior teeth. Stress distribution was investigated along and between the post and the surrounding structures. In comparison to a metal post, the most uniform distributions with lower stress values and no significant stress concentration were found when using the multilayer post.

## 1. Introduction

The role of computer-aided design (CAD) for theoretical and experimental analyses has been widely used for different applications [1,2,3,4,5,6]. Such methods have been used to develop several kinds of polymeric and composite devices and have received considerable attention in biomedical applications [7,8,9,10].

The restoration of endodontically treated teeth represents a challenge as it generally involves the use of both metals and non-metallic materials [11]. In this field, many dental post-core systems have been used [11,12]. Initially, metal posts were chosen due to their long-term safety. As a consequence of mismatch between the elastic modulus of metal alloys and the surrounding structures, stress concentration generally occurs, often leading to catastrophic root fracture [12]. For this reason, studies have been devoted to the development of different shapes, sizes, and materials for the post [12].

Considering the results of previous studies, the use of materials with a lower elastic modulus, such as fiberglass-reinforced composites, may provide more favorable stress distribution. However, these composite posts have an elastic modulus, often ranging from 45.7 to 53.8 GPa [12,13], that is lower than that of metal posts, e.g., 95 GPa for gold and 110 GPa for titanium [12,14], but is still higher than those of natural tissues, which is 18.6 GPa for dentin [12,15].

Studies on endodontically treated canine teeth showed interesting results in terms of stress distribution, focusing on the ferrule effect and on the role of the specific material-shape combination of the post [16].

The mechanical behavior of a restored tooth is negatively affected by a dental post created using a high modulus material [11]. A dental post should stabilize the core without weakening the root [11]. As reported in the literature [11], stress concentration generally occurs at the apical and cervical regions of the tooth. Thus, an ideal post would possess a stiffness that decreases from the coronal part to the apical end, optimizing the stress transfer mechanism [11]. Given this context, functionally graded materials have also been considered for the development of dental posts with tailored properties, to overcome the drawbacks related to the use of both flexible and rigid posts [11].

Titanium [17], poly(ε-caprolactone) (PCL) [9,10,18,19,20,21], and several organic-inorganic hybrid materials obtained via sol-gel method [22,23,24,25,26,27,28,29,30,31] have been proposed for different biomedical applications. For example, titanium dioxide (TiO_2_) and TiO_2_/PCL hybrid materials containing PCL up to 24% by weight were obtained using the sol-gel method. In this case, heat and pressure were applied for powder compaction. The effects of the processing conditions and the amount of polymer on the performance of the materials were properly evaluated [17].

In this study, we theoretically design a multilayer dental post with a stiffness decreasing from the coronal part to the apical end using a CAD-based approach and sol-gel chemistry. In particular, a multilayer post with a compositional gradient of sol-gel synthesized materials and a Young’s modulus ranging from 12.4 to 2.3 GPa in the coronal-apical direction was designed according to the values experimentally obtained [17] for TiO_2_/PCL 94/6 (12.4 GPa), TiO_2_/PCL 88/12 (9.2 GPa), TiO_2_ (4.1 GPa), and TiO_2_/PCL 76/24 (2.3 GPa). In endodontically treated canine teeth, the stress distribution along the multilayer post and at the interface between the post and the surrounding structure was assessed and compared to that of a titanium post. The null hypothesis was that the proposed multilayer post with a compositional gradient and a Young’s modulus varying in the coronal-apical direction would not affect the stress distribution.

## 2. Materials and Methods

### 2.1. Materials and Post

A titanium post (post A) was used as the control. TiO_2_ and TiO_2_/PCL hybrid materials containing PCL up to 24% by weight were obtained via sol-gel method as described in a previous study [17].

As the experimentally-obtained values of the Young’s modulus and Poisson’s ratio for these materials (12.4 GPa and 0.27 for TiO_2_/PCL 94/6; 9.2 GPa and 0.30 for TiO_2_/PCL 88/12; 4.1 GPa and 0.27 for TiO_2_; and 2.3 GPa and 0.30 for TiO_2_/PCL 76/24) [17], a multilayer post with a compositional gradient and a modulus varying from 12.4 to 2.3 GPa in the coronal-apical direction (post B) was designed, analyzed, and compared with a titanium post (post A) hypothetically having the same geometrical characteristics.

The geometrical characteristics of the posts are reported in Table 1.

### 2.2. Generation of the Tooth Solid Model

An upper canine was analyzed using a micro-CT scanner system (Bruker microCT, Kontich, Belgium). Micro-CT scan images were obtained and the three-dimensional (3D) CAD model of the tooth was generated as in a previous study [16], where a total of 951 slices were collected (1024 × 1024 pixels) and 252 slices were used. To process the image data sets, ScanIP^®^ (3.2, Simpleware Ltd., Exeter, U.K.) was used. A previously adopted approach was used to generate the 3D model [16]. Briefly, procedures related to image segmentation and filtering were used, and the 3D tessellated model was created [16]. Blending operations were performed via converting cross sections of the tessellated models into surfaces. ScanTo3D^®^ (SolidWorks^®^ 2017, Dassault Systemes, Paris, France) was used to manage the tessellated geometry. Specific procedures were used to create lofting surfaces and to ensure the congruence of interfacial boundaries of tooth tissues [16]. The system of coordinates, the geometrical model, and features were previously reported [16].

Two different geometric models of the restored tooth were analyzed. Specifically, two posts were considered: posts A and B (15 mm in length) with a conical-tapered shape. A 0.1 mm thick cement layer was added between the abutment and crown. In the canal, the cement was added between the post and the root. In addition, the periodontal ligament with a thickness of 0.25 mm was modelled around the root [16].

### 2.3. Numerical Simulation

The geometric models of endodontically treated anterior teeth were imported into HyperMesh^®^ (HyperWorks^®^-14.0, Altair Engineering Inc., Troy, MI, U.S.).

Finite element (FE) analyses were performed on two models: (1) Model A (a tooth with Post A) and (2) Model B (a tooth with Post B). The values of Young’s modulus and Poisson’s ratio for the components of the tooth model are reported in Table 2.

**Figure 1:**
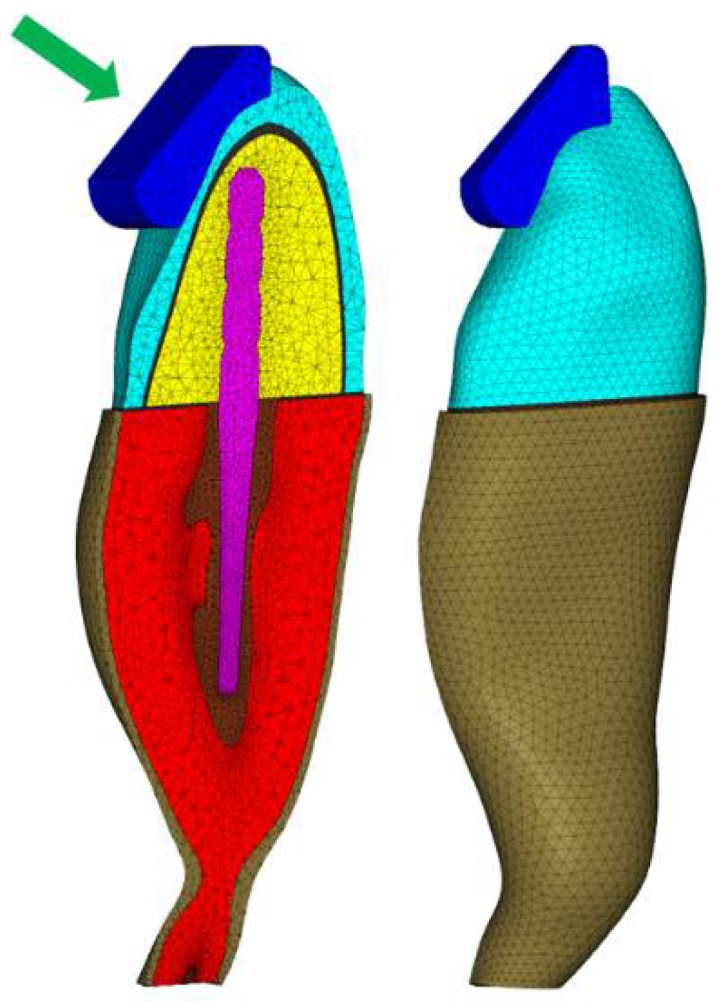
Finite element (FE) model according to the components in the geometric model.

As previously reported [16], a 3D mesh was created and 3D solid CTETRA elements with four grid points were considered for the models. Consistent with a previous methodology [16], the study focused on the closing phase of the chewing cycle and solid food acting on the crown surface, using apple pulp with a Poisson’s ratio and Young’s modulus of 0.10 and 3.41 MPa, respectively (Figure 1). Slide-type contact elements were considered between the food and tooth surface. For the contact condition between each part of the post restoration, the “freeze” type was used.

Briefly, convergence and mesh independence studies were also performed to obtain accurate results. Mesh convergence was performed to determine the number of elements needed in the model to ensure that the results were not affected by varying the mesh size. The complexity of the model vs. response (i.e., stress) was recorded. Following convergence, mesh refinement was performed. Thus, further technical features of the analyzed models included the total number of grids (structural) (51,552), elements excluding contact (213,361), node-to-surface contact elements (14,094), and degrees of freedom (188,127).

With regard to nodal displacements, the FE models of the restored tooth constraints were applied in all the directions on the surface of the periodontal ligament. On the surface of the crown, a load of 50 N was applied at 45° to the longitudinal axis of the tooth [16]. As a linear elastic behavior was assumed for all the components, a non-failure condition was considered and linear static analyses were performed. The maximum principal stress and von Mises stress distributions were evaluated along the post and at the interface between the post and the surrounding structure.

## 3. Results

**Figure 2:**
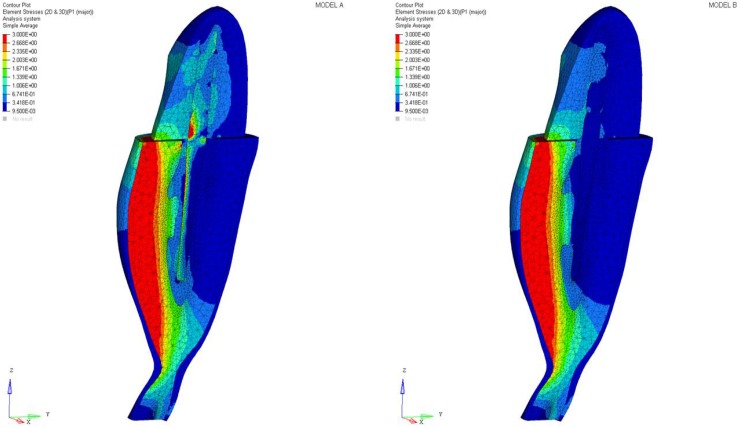
Maximum principal stress distribution (MPa): model A and model B.

**Figure 3:**
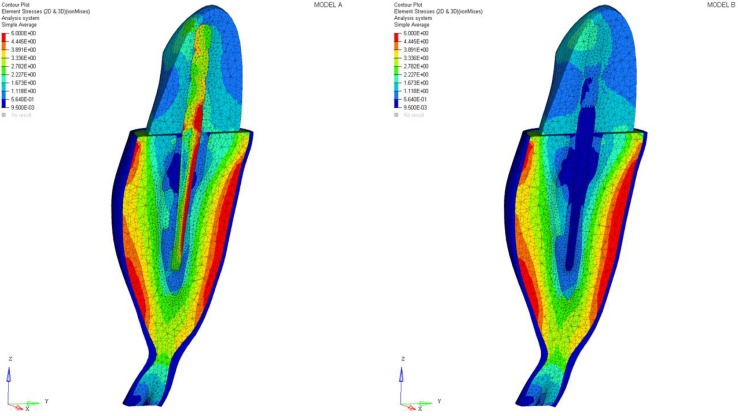
Von Mises stress distribution (MPa): model A and model B.

The maximum principal stress and von Mises stress distributions were observed in the abutment, post, post cement, root, and periodontal ligament. The considered cross sections are depicted in Figure 2 and Figure 3.

**Figure 4:**
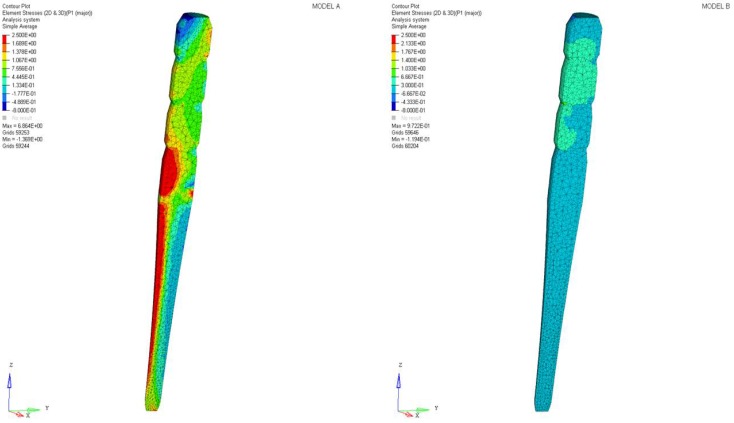
Maximum principal stress distribution (MPa): post A and post B.

**Figure 5:**
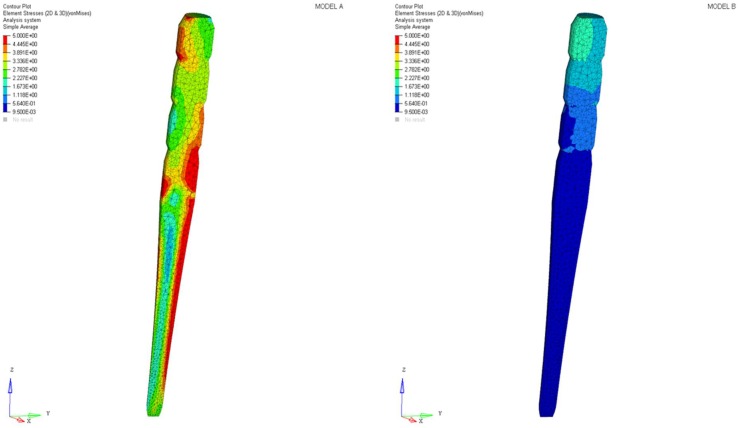
Von Mises stress distribution (MPa): post A and post B.

**Figure 6:**
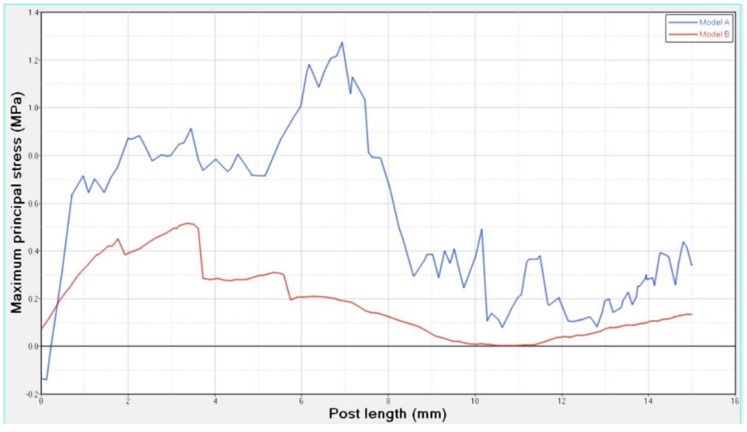
Maximum principal stress distribution along the center of the post from the coronal to the apical part.

**Figure 7:**
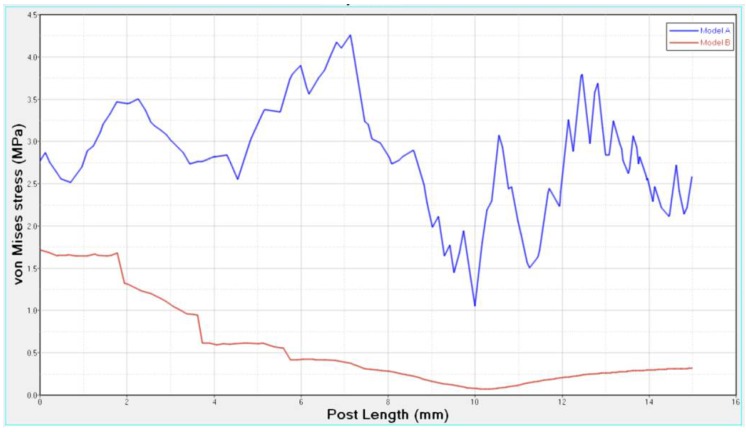
Von Mises stress distribution along the center of the post from the coronal to the apical part.

Differences were found between the two models in terms of the maximum principal stress and von Mises stress distributions. If compared to model B, higher stress regions were observed for model A along the post near the cervical margin of the tooth. Comparing the analyzed models, the most uniform stress distribution was achieved for post B. The maximum principal stress and von Mises stress distributions along the post are displayed in Figure 4, Figure 5, Figure 6 and Figure 7.

**Figure 8:**
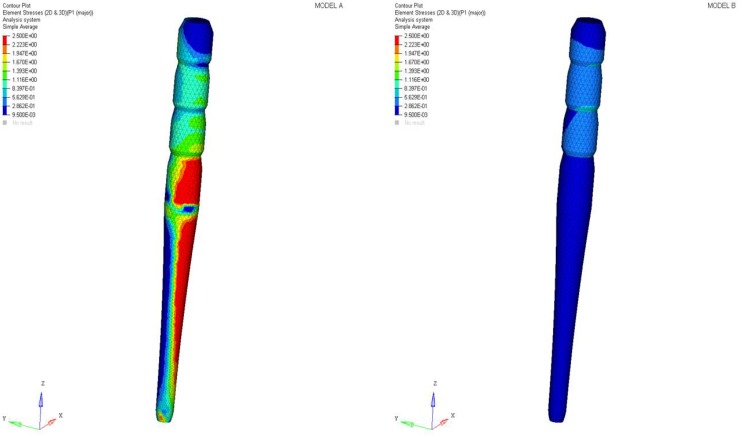
Maximum principal stress distribution (MPa) at the interface between the post and surrounding structures.

**Figure 9:**
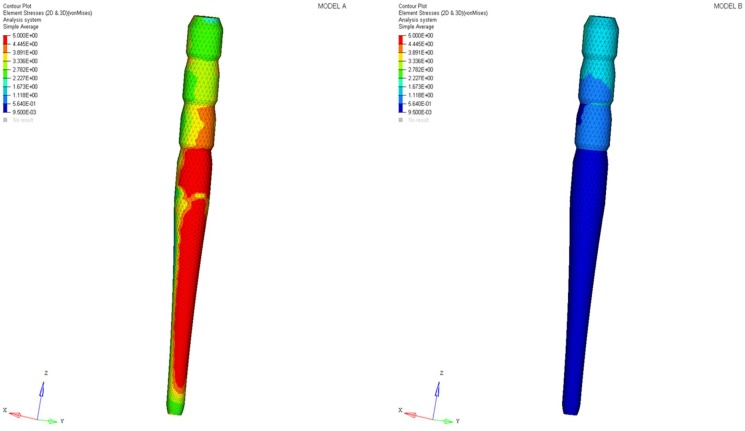
Von Mises stress distribution (MPa) at the interface between the post and surrounding structures.

**Figure 10:**
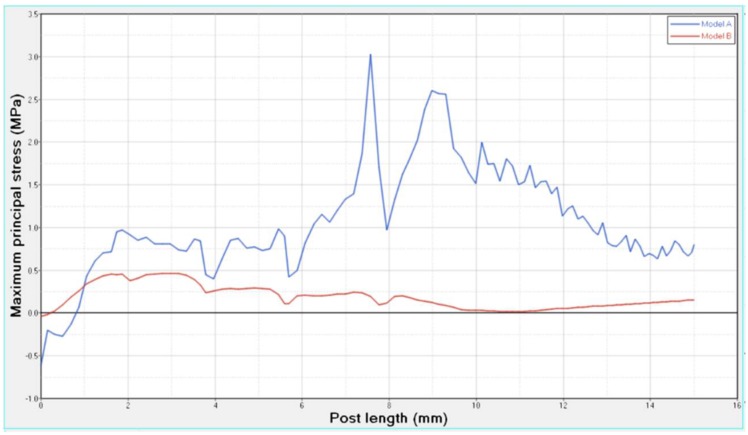
Maximum principal stress distribution at the interface between the post and surrounding structures from the coronal to the apical part.

**Figure 11:**
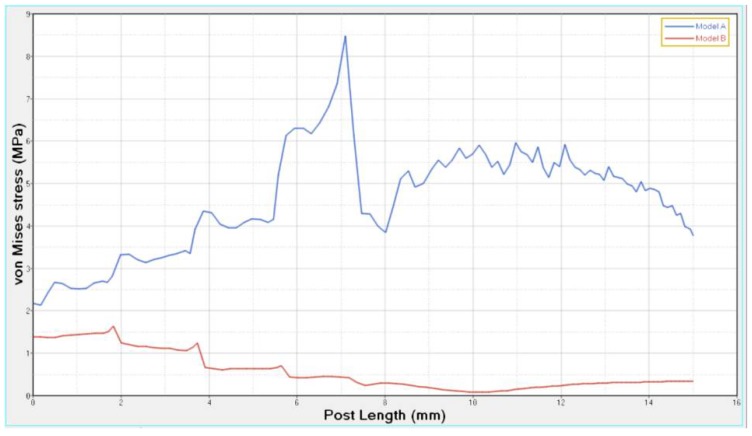
Von Mises stress distribution at the interface between the post and surrounding structures from the coronal to the apical part.

Stress concentrations were observed along the post in model A, whereas lower stress values were evident for model B. In addition, with regard to the stress distribution at the interface between the post and the surrounding structures (Figure 8, Figure 9, Figure 10 and Figure 11), for model A, high stress gradients were found as well as fluctuations and changes up to the apical part, which were much more marked than in model B.

**Figure 12:**
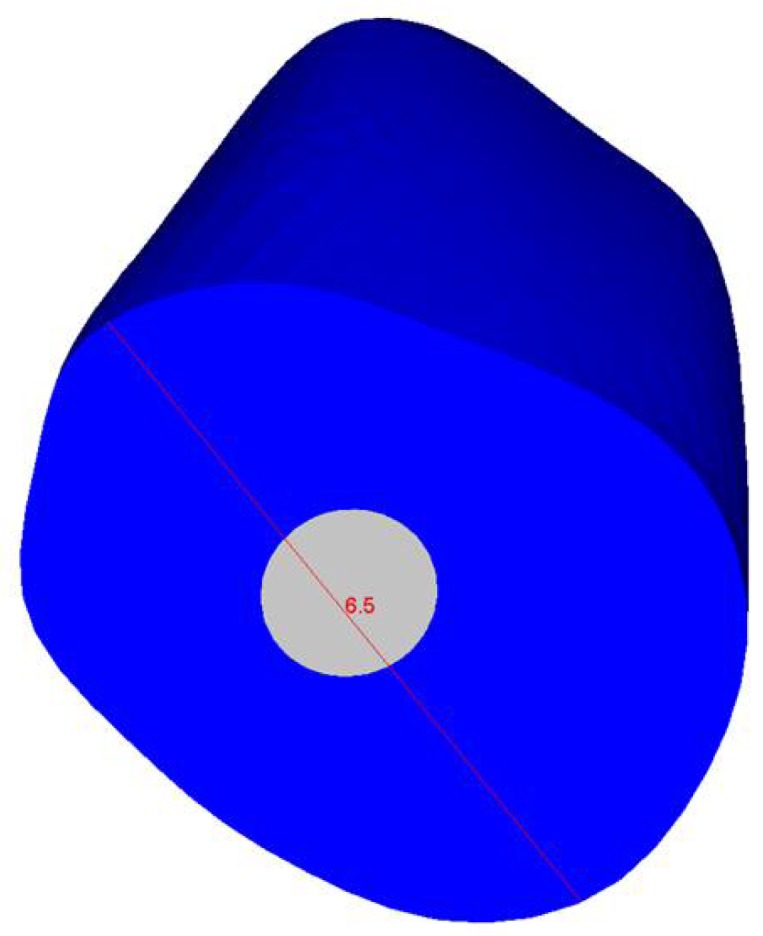
Cross section at the cervical margin of the tooth that was further analyzed.

**Figure 13:**
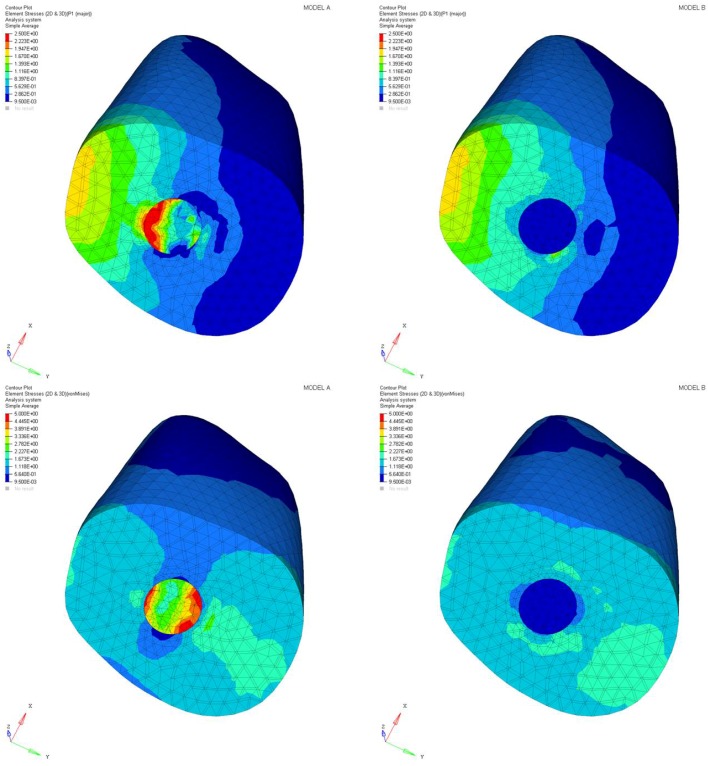
Maximum principal stress and von Mises stress distributions (MPa) in the cross-section at the cervical margin—scheme of Figure 12.

**Figure 14:**
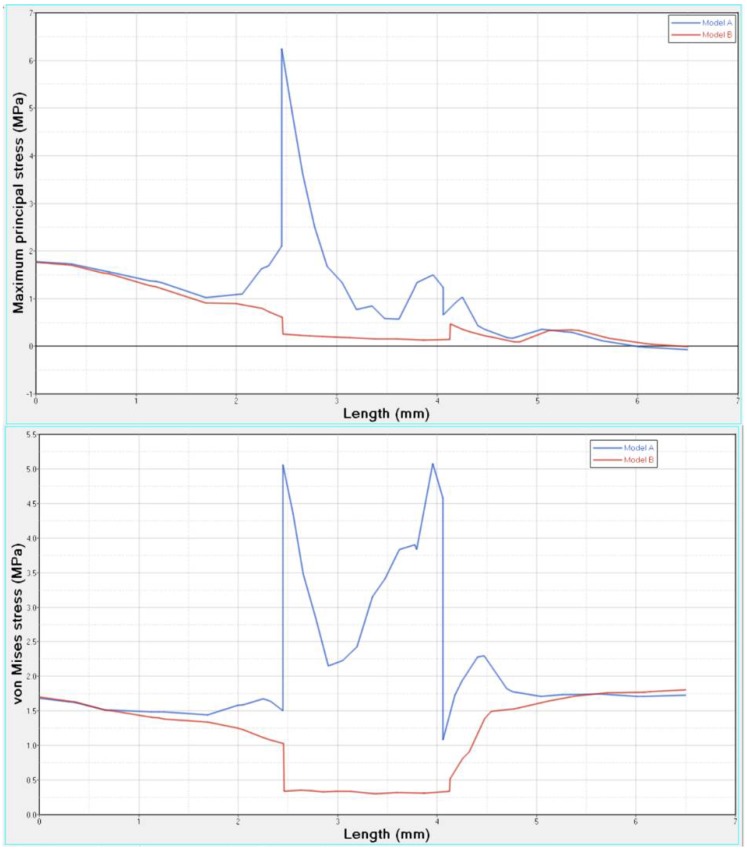
Maximum principal stress and von Mises stress distributions in the cross-section at the cervical margin, along the direction indicated by the red line shown in Figure 12.

In comparison to model A, model B showed gradual changes and lower stress values (Figure 8, Figure 9, Figure 10 and Figure 11). The differences between a cross section at the cervical margin of the tooth of the two models were compared. The results are displayed in Figure 12, Figure 13 and Figure 14.

In particular, Figure 13 and Figure 14 report the maximum principal stress and von Mises stress distributions in the cross-section at the cervical margin along the direction indicated by the red line in Figure 12. The obtained results demonstrate high stress gradients for model A at the interface between the surrounding structure and the post.

## 4. Discussion

A dental post designed using a high-modulus material clearly alters the mechanical behavior of the restored tooth [11,32]. To prevent catastrophic root fracture, fiberglass posts and resin cores are currently used as post-core systems [12]. The performance of post-and-core systems have been widely investigated [33]. As many efforts have been made to develop composite posts using different shapes and kinds of fibers, such as carbon, glass, and quartz, clinical procedures have been continuously modified [34,35,36]. Although many experimental and theoretical analyses and clinical studies have been completed, no precise recommendations have been made [35]. A general procedure includes selection of the post, the preparation of the root canal, the use of adhesive resin cements or self-adhesive cements to bond the post, which must suitably extend to retain the core, and the placement of a crown [35]. However, with regard to devices, materials, and clinical procedures, contradictory opinions still remain [35,37]. The performance of the fiber posts depends on the manufacturing process, matrix, fiber properties, distribution and amount of fibers [37]. Several clinical studies have also been performed on patients with teeth restored using posts fabricated from carbon fiber-, quartz fiber-, or glass fiber-reinforced composites [37,38,39,40,41,42].

During loading, a high stress concentration normally occurs at the apical part of the post [11,43]. When the tooth structure is compromised, an increase in flexure may cause stress concentration at the cervical region. Furthermore, stress concentration should be ascribed to the tapering of the root canal at the apical region as well as to the characteristics of the post [11,44]. High stress concentrations arise from the stiffness mismatch between the post and surrounding structures [11,45]. An ideal post should possess a stiffness decreasing from the coronal to apical part to optimize stress distribution.

As many technical features related to the development of fiber-reinforced composite posts have been widely discussed in the literature, a CAD-based approach and sol-gel chemistry were considered in the current research to theoretically design a multilayer post with a stiffness decreasing from the coronal part to the apical end.

Sol-gel chemistry has been proposed as a method to develop organic-inorganic hybrid materials with specific properties for biomedical applications [22,23,24,25,26,27,28,29,30,31]. Thus, benefiting from previous experimental results [17], TiO_2_ and TiO_2_/PCL hybrid materials containing PCL up to 24% by weight obtained using the sol-gel method were used to design a multilayer dental post with tailored properties. In particular, with regard to endodontically treated anterior teeth, the effect of a multilayer post with a compositional gradient of sol-gel synthesized materials and a Young’s modulus ranging from 12.4 to 2.3 GPa in the coronal-apical direction was evaluated in this study.

As a result of the multilayer structural design for post B, the performed analyses evidenced that higher values of maximum principal and von Mises stresses were found along the post near the cervical margin of the tooth for model A compared with model B, which showed no stress concentration (Figure 2 and Figure 3). The multilayer structure, having different mechanical properties, allowed us to tailor the performance in the coronal-apical direction and avoid stress concentration, thus providing a better stress distribution in the restored tooth. Figure 6 and Figure 7 confirm that the designed multilayer post (post B) provided better stress distribution along the center of the post from the coronal to the apical part, if compared to the titanium post (post A).

At the interface between the surrounding structures and the post, the maximum principal stress and von Mises stress distributions proved the important role of the designed post (Figure 10 and Figure 11). In the case of the titanium post, the stress transfer mechanism involved higher values of stress as well as much more marked fluctuations and changes that were evident up to the apical part (Figure 10 and Figure 11). Consistently, the analysis results of a cross section at the cervical margin of the tooth showed stress gradients for model A that were higher than those observed for model B (Figure 13 and Figure 14). Finally, the null hypothesis that the proposed multilayer post with a compositional gradient and a Young’s modulus varying in the coronal-apical direction in the restored model would not affect the stress distribution was rejected.

Potential limitations include the linear static analyses performed considering a non-failure condition and the approach used to design of the multilayer post, which was based on the results obtained in a previous work [17]. Regardless of these shortcomings, the current study should be considered as a first work toward the theoretical design of a multilayer dental post consisting of TiO_2_ and TiO_2_/PCL hybrid materials obtained using sol-gel method, with a compositional gradient and a Young’s modulus varying in the coronal-apical direction.

## 5. Conclusions

Within the limitations of the present study, the following conclusions were drawn: (1) A theoretical design of a multilayer dental post was reported using CAD-based approach and sol-gel chemistry; (2) a model of an anterior tooth restored with a multilayer post, consisting of TiO_2_ and TiO_2_/PCL hybrid materials obtained via sol-gel method, was analyzed; and (3) in comparison to a titanium post, the most uniform stress distribution with no significant stress concentrations was found in the proposed multilayer dental post with a compositional gradient and a Young’s modulus varying in the coronal-apical direction.

## Figures and Tables

**Table 1:** Geometrical characteristics of the posts: length of coronal part, length of conicity part, coronal diameters, and apical diameters.

Total Length (mm)	Length of Coronal Part (Cylindrical) (mm)	Length on Conicity Part (mm)	Coronal Diameter	Apical Diameter
15 mm	7 mm	8 mm	Ø 1.05–Ø 1.25–Ø 1.45	Ø 0.55–Ø 0.75–Ø 0.95

**Table 2:** Young’s modulus and Poisson’s ratio for the components of the tooth model [16]. * The values varied from the coronal to the apical part of the part according to the different regions [17] of the proposed multilayer post.

Component	Young’s Modulus (GPa)	Poisson’s Ratio
Lithium disilicate crown	70	0.30
Crown cement	8.2	0.30
Abutment	12	0.30
Post A	110	0.35
Post B	12.4–2.3 *	0.27–0.30 *
Post cement	8.2	0.30
Root	18.6	0.31
Periodontal ligament	0.15 (×10^−3^)	0.45
Food (apple pulp)	3.41 (×10^−3^)	0.10
